# The 7 C Vaccination Readiness Scale: An Empirical Country Comparison Between Germany and Greece

**DOI:** 10.1007/s44197-025-00485-9

**Published:** 2025-11-21

**Authors:** Birgit Teichmann, Ioannis Ladas, Demosthenes B. Panagiotakos

**Affiliations:** 1 Network Aging Research, Heidelberg University, Bergheimer Str. 20, Heidelberg, 69115 Germany; 2 Hellenic Open University, Patras, Greece; 3 Open University of Cyprus, Nicosia, Cyprus; 4 School of Health Sciences and Education, Harokopio University, Athens, Greece

**Keywords:** Vaccination readiness, Psychometrics, Vaccine acceptance, Health prevention, Confidence, Collective responsibility, Public health

## Abstract

**Background:**

Vaccinations are among the most effective interventions against infectious diseases such as measles, influenza, and COVID-19. Nevertheless, there are significant differences in vaccination readiness. The aim of this study was to validate the 7 C scale, which measures seven psychological factors comprising general vaccination readiness, in both German and Greek, and to investigate differences in vaccination behavior between a German and a Greek sample.

**Methods:**

A cross-sectional study was conducted with a convenience sample of 576 study participants, of whom 319 responded to the online survey in German and 257 in Greek. Analyses included internal consistency, structural validity, construct validity through the known-groups method, item analysis, and floor and ceiling effects. The two samples differed significantly in terms of demographic data such as age, gender and education, as well as in religiosity and vaccination behavior.

**Results:**

The German sample showed a higher vaccination rate for most vaccinations, except for vaccinations against hepatitis A, varicella, and meningococcal disease, wheras in the Greek sample, there were significantly more “I don’t know” responses regarding vaccination status. The 7 C scale of vaccination readiness demonstrated acceptable to good psychometric properties in terms of both internal consistency and construct validity in both samples, although there were some weaknesses in the component that measures *calculation*^*R*^ in both the 21-item and 7-item versions. The 7-component structure was confirmed using a confirmatory factor analysis. The German and the Greek samples differ primarily in the components *confidence*,* complacency*^*R*^, and *conspiracy*^*R*^, with significantly higher values in the German sample. Binary logistic regression analysis demonstrated that the 7 C scale had the lowest predictive value for measles vaccination and the highest for COVID-19.

**Conclusion:**

The German and Greek versions of the 7 C scale are invaluable research tools for investigating vaccination readiness. A comprehensive understanding of the underlying factors contributing to vaccine hesitancy is imperative for the development of culturally tailored educational initiatives. These initiatives must be designed to address prevalent misconceptions regarding vaccination, with the objective of enhancing vaccination rates, and promoting public health.

**Supplementary Information:**

The online version contains supplementary material available at 10.1007/s44197-025-00485-9.

## Background

Vaccination is the most important tool for the primary prevention of many serious, potentially fatal infectious diseases and one of the most cost-effective public health interventions [1]. The World Health Organization (WHO) defines vaccinations as “one of the best health investments money can buy” [2]. In addition to their conventional application in the prevention of infectious diseases, vaccines have also been utilized in the battle against tumors caused by viruses. These include, for example, cervical cancer [3] and liver carcinoma [4]. In addition, monoclonal antibodies are being developed against the tumor cells themselves [5] or for the treatment of diseases such as Alzheimer’s dementia [6] and giant cell carcinoma [7].

Every year, 3.5 to 5 million deaths worldwide are prevented by vaccinations against diseases such as diphtheria, tetanus, pertussis, influenza, and measles [2]. Although the scientific and medical consensus on the benefits of vaccination is clear and unequivocal, a growing number of people perceive vaccinations as unsafe and unnecessary [8]. The WHO has identified “hesitancy or refusal to vaccinate when vaccines are available” as one of the top ten threats to global health in 2019 [9].

Vaccine skepticism has always existed, but it has been fueled by global vaccine controversies over the past 40 years [8]. In the 1980s, for example, there were allegations that the ingredient in the pertussis vaccine caused severe brain damage, seizures, and mental retardation [10]. In the late 1990s, there were reports of an alleged link between the measles, mumps, and rubella (MMR) vaccine and autism [11], and in the 2010s, alleged adverse events associated with the HPV vaccine were reported [12]. In the period of the 2020–2023 coronavirus pandemic [13], novel mRNA vaccines were developed [14], thereby re-igniting vaccine controversies. Therefore, anti-vaccine misinformation did not just emerge with the pandemic. It has been around for a long time [15–17]. However, the internet and social media have allowed misinformation to spread rapidly, leading to the formation of online anti-vaccination organizations [18].

The success of vaccination has led to a decline in the prevalence of infectious diseases that were previously associated with high mortality rates [19], particularly in the western world where vaccines are readily available [20]. As a result, the diseases are no longer seen as threatening, which has had a major impact on people’s willingness to be vaccinated [21]. This phenomenon resulted in suboptimal vaccination coverage rates in both developed and developing countries despite the availability of vaccines [22]. Consequently, these regions became susceptible to temporary outbreaks of preventable diseases, underscoring the critical importance of effective vaccination programs in maintaining public health [23]. The COVID-19 pandemic has brought vaccination back into focus, but concerns about the safety of a vaccine developed in a rush [24] and against new vaccines like mRNA vaccines in general [25] have once again led to both misinformation and vaccination skepticism.

While estimates suggest that less than 5–10% of the population is firmly opposed to vaccination [26], a larger proportion could be considered vaccine hesitant [27]. Vaccine hesitancy, defined as delayed acceptance or refusal of vaccination despite the availability of vaccine offers [28], is complex and context-specific, varying by time, place and vaccine [29]. It can be described on a continuum ranging from individuals who have complete confidence in vaccines to those who reject them [28].

The decision to be vaccinated is usually based primarily on the subjectively expected benefit-harm ratio for one’s own health, whereby both protection against infections and vaccination reactions must be taken into account. Individual risk-related personal characteristics (e.g., age, chronic diseases, professional contacts) are decisive both for the individual’s decision and for official recommendations [30]. These risk indicators contribute to the systematic variance in the population’s willingness to be vaccinated. In general, a valid assessment of the willingness to vaccinate must, therefore, take into account the objective threat of potential infection, individual susceptibility to infection, and subjective perception of the threat in an integrated manner [31]. Nevertheless, the causes of vaccination hesitancy are insufficiently researched, with arguments ranging from scientifically sound data to conspiracy theories [32].

The aforementioned context-specific nature of vaccine hesitancy has led to the development of numerous instruments to measure vaccine hesitancy. Betsch et al. (2018) developed an instrument to measure five key psychological facets of vaccine hesitancy, namely confidence, constraints, complacency, calculation and collective responsibility, which is also referred to as the 5 C scale [33]. While *confidence* reflects trust in the effectiveness and safety of vaccination, the healthcare system, and the motives of decision makers, *complacency* describes the perception of health risks that should be avoided by vaccination and whether vaccination is considered necessary. The component *constraints* refers to barriers to vaccination uptake, such as effort, stress, and availability, and whether vaccinations are considered sufficiently important to protect against health barriers. Finally, *calculation* refers to an active search for information accompanied by a conscious risk-benefit assessment. A low level of calculation indicates an disregard for cost-benefit considerations about vaccinations and a high level of vaccination readiness. *Collective responsibility* indicates the extent of prosocial motivation to be vaccinated. It is considered beneficial to vaccinate oneself, as this reduces the risk for other individuals within one’s family or community, particularly in contexts where herd immunity is feasible.

In light of the recent corona pandemic, Geiger et al. (2022) have augmented the 5 C scale with two additional factors: *conspiracy*, given its status as one of the most pervasive and extensively endorsed medical conspiracy theories, and *compliance*, aimed at assessing an individual’s adherence to vaccination policies, that is, the degree to which an individual endorses the measures implemented by states to address pandemics [34].

The aim of this study is to validate the 7 C scale of vaccination readiness in German and Greek, while also comparing vaccination readiness in Germany and Greece. The rationale of comparing these two countries has to do with the differing sociocultural, political, and public health contexts that may influence attitudes toward vaccination. Germany and Greece vary significantly in terms of healthcare infrastructure, vaccine policies, and historical experiences with vaccine campaigns. By comparing these two countries, the study explores how these contextual factors impact the components of vaccination readiness as measured by the 7 C scale, and to assess the scale’s cross-cultural validity and applicability in diverse European settings. In particular, it is being investigated whether there are differences in the willingness to be vaccinated regarding different vaccines. On the one hand, the measles vaccination is examined, for which two doses are sufficient for lifelong immunity and which is usually given in childhood [35], on the other hand the vaccination against influenza and COVID-19, both of which are recommended as an annual vaccination, especially for people over 60 years of age [36]. While conventional vaccines are used for the influenza vaccination, which can be designed as dead or live vaccines, standard-dose or high-dose, egg or cell culture-based, with or without adjuvant [37], modern vaccines are available for the COVID-19 vaccination, especially mRNA vaccines [38].

## Methods

### Participants and Data Collection

Between July 2024 and February 2025, a cross-sectional online survey was conducted using the SoSci Survey platform. The anonymous online survey was made available to participants at www.soscisurvey.de [39]. Information about the study was communicated through various channels, including the website of the Network Aging Research (NAR) at Heidelberg University, flyers, newsletters, posters, and social media such as WhatsApp, Instagram, and Facebook. In total, data were collected from *N* = 576 individuals, of which *n* = 319 responded to the German version of the questionnaire and *n* = 257 responded to the Greek version.

To ensure sufficient statistical power, we performed a power analysis using the software G*Power 3.1.9.7 [40] according to Kang’s guidelines [41]. For the country differences, an effect size of at least d = 0.5 was anticipated, with a targeted power of 1 - β = 0.95 and a maintained significance level of α = 0.05. In accordance with the recommendation of G*Power, it is deemed appropriate to establish a total sample size of *N* = 176, which is equivalent to *n* = 88 for each group. Given the necessity to conduct both t-Tests for the country comparison and t-Tests to verify construct validity within a country, the total sample size required is *n* = 176 persons per country.

### Questionnaire Design

Participants provided information about their demographics, religiousness, vaccination status, intention to be vaccinated against measles, influenza, and COVID-19, along with their answers to the 7 C scale of vaccination readiness [34]. In addition, two scales were used, which are not taken into account in this study, the Consequentialist Scale [42] and the Big Five Inventory-10 [43]. The order of the scales was predetermined, but the questions within the scales were randomized. The questionnaire was estimated to require approximately 10–15 min to complete. We provide the Greek and German versions as well as the data of the study on Open Science Framework (OSF, https://osf.io/ynhe4/).

#### Demographic Characteristics

The survey requested respondents’ sociodemographic data, including gender, age, country of residence, educational qualifications, employment status, occupation, income, marital status, number of children, and religiousness. The degree of religiousness was measured using the following three questions: To what extent does religion play a role in your life? What is the frequency with which you partake in religious services? To what extent do religious beliefs influence personal decision-making processes? The initial question and the third question could be addressed through the utilization of a Likert scale, ranging from 1 (not at all) to 5 (very much), while the question concerning the frequency of church attendance could be addressed through a 6-point scale ranging from 1 (not at all) to 6 (daily). The total score obtained from the summation of the three questions ranged from 3 to 16. Furthermore, participants were requested to evaluate their physical and mental well-being employing a 5-point Likert scale ranging from 1 (very good) to 5 (very poor) and to appraise their life satisfaction utilizing a 10-point Likert scale from 1 (extremely unsatisfied) to 10 (extremely satisfied). Additionally, participants were asked to specify their professional affiliation, indicating whether their work was in the medical, therapeutic, or care sector and whether it involved direct interaction with vulnerable individuals.

#### Reasons for Vaccination

Regarding the reasons for vaccination, participants were invited to explain their decision to vaccinate. Specifically, they were asked whether this was a conscious decision and, if so, which factors influenced them. Participants were asked about the measles vaccination, specifically if their parents had made the decision, since it is usually administered in childhood and provides lifelong protection with two doses. In relation to the matter of vaccinations, participants were also invited to provide a rationale for their decision to vaccinate, i.e., whether this was a conscious decision and, if so, what the reasons for this were. Participants were therefore asked about the measles vaccination and whether the decision had been made by their parents, as this is usually given in childhood, whereby two doses provide lifelong protection. For the flu and COVID vaccinations, participants were asked whether the vaccination had been recommended by a doctor, as these are recommended annually for certain groups. They were also asked to what extent they had concerns about vaccination, whether they felt they belonged to a risk group, or whether they were getting vaccinated to protect family members or friends. There was also an option to specify other reasons in a text field.

#### Vaccination Readiness Scale

The 7 C scale of vaccination readiness consists of 21 items, three items for each of the seven components, namely *confidence*, *complacency*^R^, *constraints*^R^, *calculation*^R^, *collective responsibility*, *compliance*, and *conspiracy*^R^, answered on a seven-point Likert scale ranging from 1 (strongly disagree) to 7 (strongly agree). Nine items were reverse-coded (4, 5, 9, 10, 11, 12, 19, 20, 21) [34]. Following Schulz et al. 2024 [31], we have labelled those components that have a reversed naming as R (reversed), i.e., high values of the component conspiracy^R^ mean that conspiracy thinking and belief in fake news is low. As proposed by Geiger et al. 2022 [34], the 7 C 21-item scale was modeled in a bifactor model with *confidence* as a reference factor and a dominant general factor “readiness to vaccinate” [44]. The 7 C scale also has a short version with one item for each of the seven components (items 3, 6, 8, 11, 14, 18, 19), which was modeled as a g-factor model. Both models fit the data acceptably in Geiger et al.’s (2022) study of Danish adults [34]. The German and Greek versions were provided by Mattis Geiger and are available online at https://vaccination-readiness.com/materials/ [45]. To the best of our knowledge, no validation study has been conducted in these languages so far, although both versions have been utilized for research purposes.

### Statistical Analysis

The descriptive and inferential statistical analysis was conducted via IBM Statistical Package for Social Sciences (SPSS) Version 29.0.1.1, while the bifactor confirmatory factor analyses (CFA) were conducted via AMOS version 29. For the cross-cultural comparison, the data of the two samples were compared using χ^2^-test for categorial data and unpaired t-Tests for continuous data. Cramer φ statistic (range 0 to 1) was calculated to evaluate the strength of the association between categorical variables (the higher, the strengthen), while for the metric variables Cohen’s d was determined to calculate the effect size, that is how much the mean values of two samples differ, with IdI = 0.20 indicates a small effect, IdI = 0.50 indicates a medium effect and IdI = 0.80 indicates a large effect [46, 47]. The means and standard deviations of the scores obtained from the 7 C scale were calculated. In addition, a histogram was constructed. The psychometric evaluation of the 7 C scale included internal consistency (Cronbach’s alpha and McDonald’s omega), structural validity (bifactor confirmatory factor analysis, g-factor confirmatory factor analysis), construct validity (employing the known-groups method), item analysis, and concurrent validity (correlation with vaccination behavior and vaccination intention). Binary logistic regression was utilized to predict vaccination intention against measles, influenza, and COVID-19.

#### Internal Consistency

For assessing internal consistency, Cronbach’s alpha was computed, which evaluates the reliability of scales comprising more than 10 items [48]. In addition, McDonald’s omega was calculated, as it accounts for varying unstandardized factor loadings and error variances, which can provide a potentially more robust measure of internal consistency [49, 50]. Values between 0.70 and 0.95 indicate good internal consistency, while values exceeding 0.95 may suggest item redundancy due to overlapping content [51].

#### Structural Validity

The 7 C scale was modeled in a bifactor model with confidence as a reference factor. In addition, the short scale with seven items, one for each category, was modeled in a g-factor model. The overall model fit was tested using the comparative fit index (CFI), the Tucker-Levis index (TLI), the root mean square error of approximation (RMSEA), and the standardized root mean square residual (SRMR). Model fit and factor saturation were deemed acceptable at CFI and TLI ≥ 0.90, RMSEA < 0.08 and SRMR < 0.11 and good at CFI and TLI ≥ 0.95, RMSEA < 0.05 and SRMR < 0.08 [52–54]. All models are estimated by a robust maximum likelihood estimation (MLR). The significance is evaluated using an alpha level of α = 0.05.

#### Construct Validity

We assessed construct validity using the known-groups method, which distinguishes two distinct groups based on expected differences in their scale scores. We formulated the following hypotheses for this study:

1. People over 60 years of age have significantly higher vaccination readiness than younger people.
2. People who work with older adults have significantly higher vaccination readiness than people who do not work with older adults.
3. People who intend to be vaccinated against influenza or COVID-19 have a significantly higher vaccination readiness than people who do not intend to be vaccinated.

#### Item Analysis

The item-total correlations are a measure of consistency between the score of an individual item and the total scale score and provide valuable insight into the explanatory power of each item. Furthermore, inter-item correlations were examined to assess the strength of the relationship between items. Typically, mean item-total and mean inter-item correlations in the range of 0.2–0.4 are considered indicative of a significant contribution of information to the scale. However, it is imperative to acknowledge that higher correlations do not inherently signify higher reliability. An excessively high correlation may be indicative of redundancy in the items, resulting in an artificial inflation of reliability [55].

#### Binominal Logistic Regression

Binominal logistic regression analyses were performed to examine whether the 21-item 7 C scale and its components are significant predictors for the intention to be vaccinated, controlling for sociodemographic variables.

## Results

### Sociodemographic Characteristics of the Participants

A total of 576 individuals participated in the study, with 319 responding to the questionnaire in German and 257 in Greek. Table 1 presents the sociodemographic data for both samples, along with the statistical differences between them.

**Table 1:** Sample characteristics for the German and the Greek samples

	German sample (*n* = 319)		Greek sample (*n* = 257)		*P* ^8^
**Age (M**,** SD)**	43.08 (18.77)		38.99 (14.27)		**.004**
**Gender (n**,** %)**					**.016**
female male diverse	230 (72.1) 84 (26.3) 5 (1.6)		168 (65.1) 89 (34.5) 0 (0.00)		
**Marital status (n**,** %)**					.608
married in partnership single divorced widowed or partner deceased	121 (37.9) 70 (21.9) 101 (31.7) 15 (4.7) 12 (3.8)		114 (44.4) 51 (19.8) 75 (29.2) 10 (3.9) 7 (2.7)		
**Children (%)**	128 (40.1)		112 (43.6)		.403
**Country**	Germany Austria Switzerland other	293 (91.8) 11 (3.4) 2 (0.6) 13 (4.1)	Greece Cyprus other	227 (88.3) 17 (6.6) 13 (5.1)	
**Education**					**< .001**
0–13 years Bachelor or Master PhD	111 (34.8) 171 (53.6) 37 (11.6)		61 (23.7) 162 (63.0) 34 (13.2)		
**Employment**					**< .001**
still in education employed unemployed/housewife, househusband retired other	47 (14.7) 190 (59.6) 7 (2.2) 58 (18.2) 17 (5.3)		12 (4.7) 186 (72.4) 20 (7.7) 5 (1.9) 34 (13.2)		
**Income (n**,** %)**					**< .001**
less than 250 € 250–500 € 500-1.000.000 € 1.000–1.500.000.500 € 1.500–2.000.500.000 € 2.000–3.000 € 3.000–4.000 € 4.000–5.000 € 5.000 € and more does not wish to answer no own income	4 (1.3) 8 (2.5) 31 (9.7) 37 (11.6) 34 (10.7) 74 (23.2) 46 (14.4) 22 (6.9) 16 (5.0) 25 (7.8) 22 (6.9)		19 (7.4) 9 (3.5) 35 (13.6) 59 (23.0) 41 (16.0) 28 (10.9) 10 (3.9) 3 (1.2) 13 (5.1) 38 (14.8) 2 (0.8)		
**Do you work in a medical/therapeutic/nursing field? (n, %)**					**< .001**
yes no I don’t know	66 (20.7) 247 (77.4) 6 (1.9)		104 (40.5) 152 (59.1) 1 (0.4)		
**Do you work with** ^1^					
children older people with other vulnerable groups no, with none of the above	44 (13.8) 45 (14.1) 25 (7.8) 224 (70.2)		48 (18.7) 57 (22.2) 31 (12.1) 152 (59.1)		.115 **.012** .091 **.005**
**Health status**					**.007**
very good good satisfied less good poor	89 (27.9) 135 (42.3) 71 (22.3) 20 (6.3) 4 (1.3)		48 (18.7) 102 (39.7) 88 (34.2) 13 (5.1) 6 (2.3)		
**Life satisfaction**^**2**^ **(M**,** SD)**	7.34 (1.90)		7.26 (1.61)		.635
**Religiousness**					
How religious are you?^4^ How often do you attend religious services? ^5^ How much does religion influence your decisions?^6^	2.07 (1.15) 2.07 (1.15) 1.83 (1.07)		2.73 (1.32) 2.40 (1.21) 1.96 (1.09)		**< .001** **.004** .810
Religiousness total^7^	5.70 (2.77)		7.09 (3.24)		**< .001**

^*1*^*More than one selection is possible.*^*2*^*Life satisfaction was assessed by a single item with a 10-point response scale from 1 = very dissatisfied to 10 = very satisfied.* ^*4,6*^*Possible answers from 1 (not at all) to 5 (very much).* ^*5*^*Attendance at church services: 1 = not at all*,* 2 = once a year*,* 3 = several times a year*,* 4 = once a month*,* 5 = once a week*,* 6 = every day.*^*6*^*Total score from the three questions on religiousness (possible values: 3–16).* ^*5*^*The significance of the differences between the German and Greek samples was determined for metric variables using a t-Test and for categorical variables using a χ*^*2*^ *test*

As the samples in both studies were convenience samples, the participants differed significantly in their demographic characteristics. The German sample was significantly older (M = 43.08 years) than the Greek sample (M = 38.99 years), and the proportion of women was significantly higher (72.1% vs. 65.1%). Greater levels of education were observed in the Greek sample, whilst a higher proportion of the sample in Germany reported being retired. The proportion of individuals who self-rated their health as “very good” was higher in the German sample (27.9% vs. 18.7%), whereas the samples did not differ in terms of life satisfaction. Although the participants in the Greek sample attend religious services significantly more often and classify themselves as more religious, their decisions are not more influenced by religion.

### Vaccination Status

Table 2 shows the vaccination status. The full table of reported vaccinations can be found in Appendix 1. Differences between the two samples were observed for the MMR vaccination (χ^2^(2, *N* = 576) = 7.112, *p* =.029, φ = 0.111), with the difference in the proportion of “Don’t know” responses being particularly striking. While 10.5% of Greek participants did not know whether they had been vaccinated, only 5.3% of German participants did not. Other significant differences in vaccination status were found for diphtheria (χ^2^(2, *N* = 576) = 22.727, *p* <.001, φ = 0.199), tetanus (χ^2^(2, *N* = 576) = 33.606, *p* <.001, φ = 0.242), poliomyelitis (χ^2^(2, *N* = 576) = 49.872, *p* <.001, φ = 0.294), hepatitis B (χ^2^(2, *N* = 576) = 8.930, *p* =.012, φ = 0.125), with more people vaccinated in Germany than in Greece. Vaccination rates for varicella (χ^2^(2, *N* = 576) = 17.533, *p* <.001, φ = 0.174), meningococcal B (χ^2^(2, *N* = 576) = 12.382, *p* =.002, φ = 0.147), meningococcal C (χ^2^(2, *N* = 576) = 13.125, *p* =.001, φ = 0.151) and hepatitis A (χ^2^(2, *N* = 576) = 102.394, *p* <.001, φ = 0.422) also differed significantly, with more people vaccinated in Greece than in Germany.

**Table 2:** Vaccination status

Vaccination type (*n*, %)	German sample (*n* = 319)	Greek sample (*n* = 257)	*p* ^1^
**Measles**,** mumps**,** rubella (MMR)**			
yes	262 (82.1)	208 (80.6)	**.02**
no	40 (12.5)	22 (8.5)	
I don’t know	17 (5.3)	27 (10.5)	
**Influenza**			
yes, one time	33 (10.3)	40 (15.6)	.266
yes, several times	84 (26.3)	53 (20.6)	
yes, every year	72 (22.6)	58 (22.6)	
no	113 (35.4)	90 (35.0)	
I don’t know	17 (5.3)	16 (6.2)	
**COVID-19**			
yes	292 (91.5)	222 (86.4)	.073 **< .001**
no	27 (8.5)	35 (13.6)	
one time	4 (1.3)	7 (2.7)	
two times	38 (11.9)	71 (27.6)	
three times and more	250 (78.4)	144 (56.0)	
I don’t know	0 (0.0)	0 (0.0)	
**Diphtheria**			
yes	236 (74.0)	142 (55.0)	**< .001**
no	17 (5.3)	29 (11.2)	
I don’t know	66 (20.7)	86 (33.3)	
**Tetanus**			
yes	306 (95.9)	208 (80.6)	**< .001**
no	5 (1.6)	24 (9.3)	
I don’t know	8 (2.5)	25 (9.7)	
**Pertussis**			
yes	208 (65.2)	154 (59.7)	.062
no	50 (15.7)	33 (12.8)	
I don’t know	61 (19.1)	70 (27.1)	
**Poliomyelitis**			
yes	277 (86.8)	158 (61.2)	**< .001**
no	10 (3.1)	29 (11.2)	
I don’t know	32 (10.0)	70 (27.1)	
**Hepatitis B**			
yes	243 (76.2)	175 (67.8)	**.012**
no	43 (13.5)	33 (12.8)	
I don’t know	33 (10.3)	49 (19.0)	
**Varicella**			
yes	123 (38.6)	144 (55.8)	**< .001**
no	155 (48.6)	88 (34.1)	
I don’t know	41 (12.9)	25 (9.7)	
**Other vaccinations**			
Hepatitis A	36 (11.3)	127 (49.4)	**< .001** **< .001**
FSME	68 (21.3)	0 (0.0)	
other	81 (25.4)	1 (0.4)	

### Reasons for Vaccination

As shown in Table 3, the decision to vaccinate against measles was mostly made by parents, whereas the main reason for vaccination against influenza and COVID-19 was to protect others. Overall, the responses differed significantly between the German and Greek samples, with χ^2^(6, *N* = 576) = 25.748, *p* <.001, φ = 0.211) for measles, χ^2^(7, *N* = 576) = 87.850, *p* <.001, φ = 0.391 for influenza, and χ^2^(7, *N* = 576) = 36.234, *p* <.001, φ = 0.251 for COVID-19.

**Table 3:** Reasons to vaccinate against measles, influenza and COVID-19

Reason	Measles *n* (%)			Influenza *n* (%)			COVID-19 *n* (%)		
	German	Greek	*p*	German	Greek	*p*	German	Greek	*p*
Parents^1^/Physician^2^	236 (74.0)	223 (86.8)	< .001	53 (16.6)	63 (24.5)	< .001	45 (14.2)	52 (20.2)	< .001
Duty	9 (2.8)	3 (1.2)		5 (1.6)	30 (11.7)		45 (14.2)	62 (24.1)	
Belonging to a risk group	0 (0.0)	0 (0.0)		53 (16.6)	3 (1.2)		10 (3.1)	2 (0.8)	
Antibodies present due to a previous illness	54 (16.9)	14 (5.4)		17 (5.3)	9 (3.5)		3 (0.9)	8 (3.1)	
Concerns about side effects	3 (0.9)	2 (0.8)		18 (5.6)	10 (3.9)		13 (4.1)	19 (7.4)	
Not belonging to the risk group	2 (0.6)	6 (2.3)		55 (17.2)	61 (23.7)		4 (1.3)	7 (2.7)	
For the protection of others	3 (0.9)	4 (1.6)		31 (29.7)	46 (17.9)		102 (32.4)	66 (25.7)	
Other reasons	12 (3.8)	5 (1.9)		87 (27.3)	35 (13.6)		95 (29.9)	41 (16.0)	

Many participants also took the opportunity to give free responses. Regarding measles vaccination, respondents in the German group noted that when they were children, no measles vaccine was available. In addition to protecting others, participants in both samples stated that they would get vaccinated for their own sake due to illness, and several participants in the German sample also cited their protection of their unborn child as a reason.

Regarding flu vaccination, participants in both samples said that it was not necessary because they were either not in a risk group or not susceptible. In the German sample, it was often said that people wanted to protect themselves because they were in contact with many people, their employer offered the vaccination, which was very convenient, or they did not want to get seriously ill. Pregnancy was also mentioned as a reason in both samples, while occupation (teachers and medical staff) was noted in the German sample.

In addition to the reasons already mentioned for influenza, the reasons given for the COVID-19 vaccination included being forced to be vaccinated to participate in social life again. One person in the Greek sample also stated that he had been vaccinated to avoid losing his job. Some participants in both samples said that the vaccination was not effective, and one person in each sample considered it to be a high-risk experiment or “an experiment in giving genetic instructions to healthy people”. Participants in the German sample said they trusted science and simply wanted to protect themselves.

### 7 C Scale of Vaccination Readiness

#### Descriptive Statistics of the 7 C Scale

Table 4 shows the means and standard deviations of the 7 C scales and the seven psychological factors, along with the differences between the Greek and German samples.

**Table 4:** Comparison between the components and the 7 C scales between the German and Greek samples

	German sample (*n* = 319) M (SD)	Greek sample (*n* = 257) M (SD)	T	df	*p*	d
Confidence	4.91 (1.56)	4.37 (1.35)	4.346	**574**	**< .001**	0.36
Complacency^R^	5.92 (1.27)	5.61 (1.11)	3.020	**574**	**.003**	0.25
Constraints^R^	5.00 (1.34)	5.10 (1.13)	− 0.926	**574**	.355	− 0.08
Calculation^R^	2.97 (1.22)	3.13 (1.25)	−1.555	**543.016**	.121	− 0.13
Collective Responsibility	5.57 (1.69)	5.69 (1.30)	− 0.974	**574**	.331	− 0.08
Compliance	3.75 (1.61)	3.94 (1.46)	−1.504	**574**	.130	− 0.13
Conspiracy^R^	5.44 (1.53)	4.72 (1.28)	8.446	**554.947**	**< .001**	0.71
C7 21-item version	4.79 (1.12)	4.65 (0.88)	1.992	**574**	**.047**	0.17
C7 7-item version	4.58 (1.26)	4.40 (1.05)	1.762	**574**	.079	0.15

^R^The components are coded so that lower values indicate higher component levels. M, mean; SD, standard deviation

The two samples differ significantly in the *confidence*, *complacency*^*R*^ and *conspiracy*^*R*^ components, with the German sample scoring higher. The effect size, as measured by Cohen’s d, was low to medium for *confidence*, low for *complacency*^*R*^, and medium to high for *conspiracy*^*R*^. This means that the German participants have greater confidence in health authorities, lower complacency and less belief in conspiracy theories. The 21-item version of the two samples differs significantly, with the German sample showing a higher readiness to be vaccinated. This is also evident in the short version, but here the difference between the two samples is not significant.

The 21-item scale correlates strongly and significantly with the 7-item version (German scale *r* =.93, *p* <.001; Greek scale *r* =.94, *p* <.001, respectively).

**Fig. 1:**
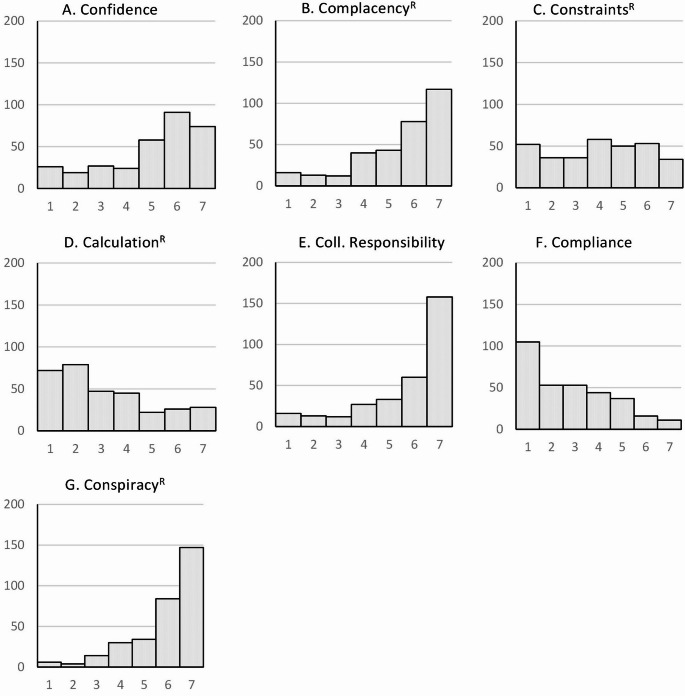
Histogram of 7 C scale scores for the German sample. The vertical axis represents the number of participants, and the horizontal axis represents the 7 C scale scores

Figure 1 shows the histograms of the German sample. Here, only *constraints*^*R*^ showed a modal value of 4, while *confidence*,* complacency*^*R*^,* collective responsibility* and *conspiracy*^*R*^ showed ceiling effects, whereas *compliance* showed a floor effect.

**Fig. 2:**
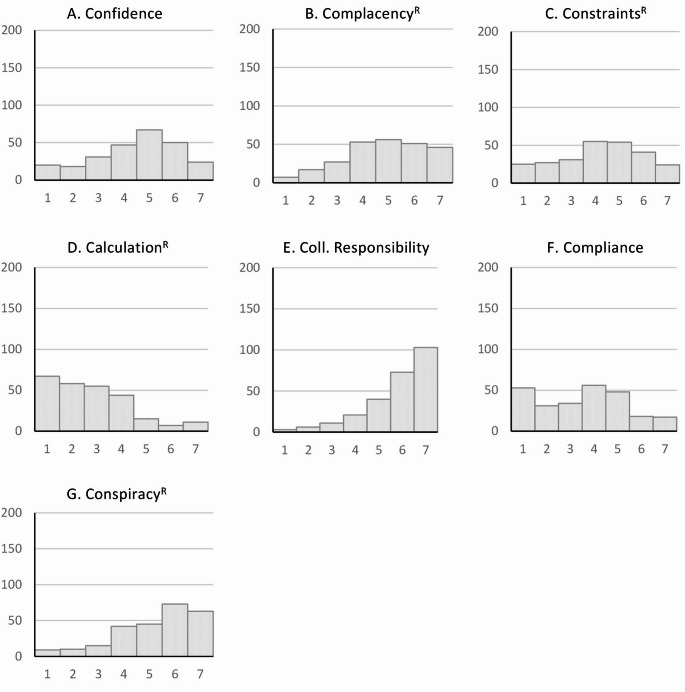
Histogram of 7 C scale scores for the Greek sample. The vertical axis represents the number of participants, and the horizontal axis represents the 7 C scale scores

In the Greek sample (Fig. 2), *constraints*^*R*^ and *compliance* showed a modal value of 4, whereas only *collective responsibility* and *conspiracy*^*R*^ showed a ceiling effect and *calculation*^*R*^ a floor effect. *Constraints*^*R*^ and *compliance* showed a modal value of 4, corresponding to a normal distribution.

####  Internal Consistency

The Cronbach’s alpha values for the total sample (α = 0.91), the German (α = 0.92), and the Greek samples (α = 0.88) are excellent for the 21-item version and good to excellent for the 7-item version (α = 0.83 and α = 0.76, respectively). McDonald’s omega was calculated for the bifactor model and yielded lower values, with omega = 0.80 for the German sample and omega = 0.65 for the Greek sample. The values for the 7-item were similar to the Cronbach’s alpha values, as a g-factor model was used, with omega = 0.85 for the German and 0.778 for the Greek sample.

#### Item Analysis

The item analysis of the C7 scale shows overall satisfactory results in both samples, with the exception of the items 10, 11, and 12 that make up the component *calculation*^*R* a^nd item 9. In particular, item 10 “*I get vaccinated when I do not see disadvantages for me*” (reverse-coded item) correlates negatively with all items except item 9 and item 11, in the Greek sample also with item 12. The Cronbach alpha would increase in both samples when these four items were deleted. All other item-total correlations exceed the recommended threshold of 0.4, in the German sample, even of 0.5.

A more thorough examination of the items reveals that there is redundancy only between item 13 *“I also get vaccinated because protecting vulnerable risk groups is important to me*” and item 15 “*I also get vaccinated because I am thereby protecting other people*” with an inter-item correlation of 0.861 for the German and 0.785 for the Greek sample, indicating that they are statistically almost identical.

#### Structural Validity

**Fig. 3:**
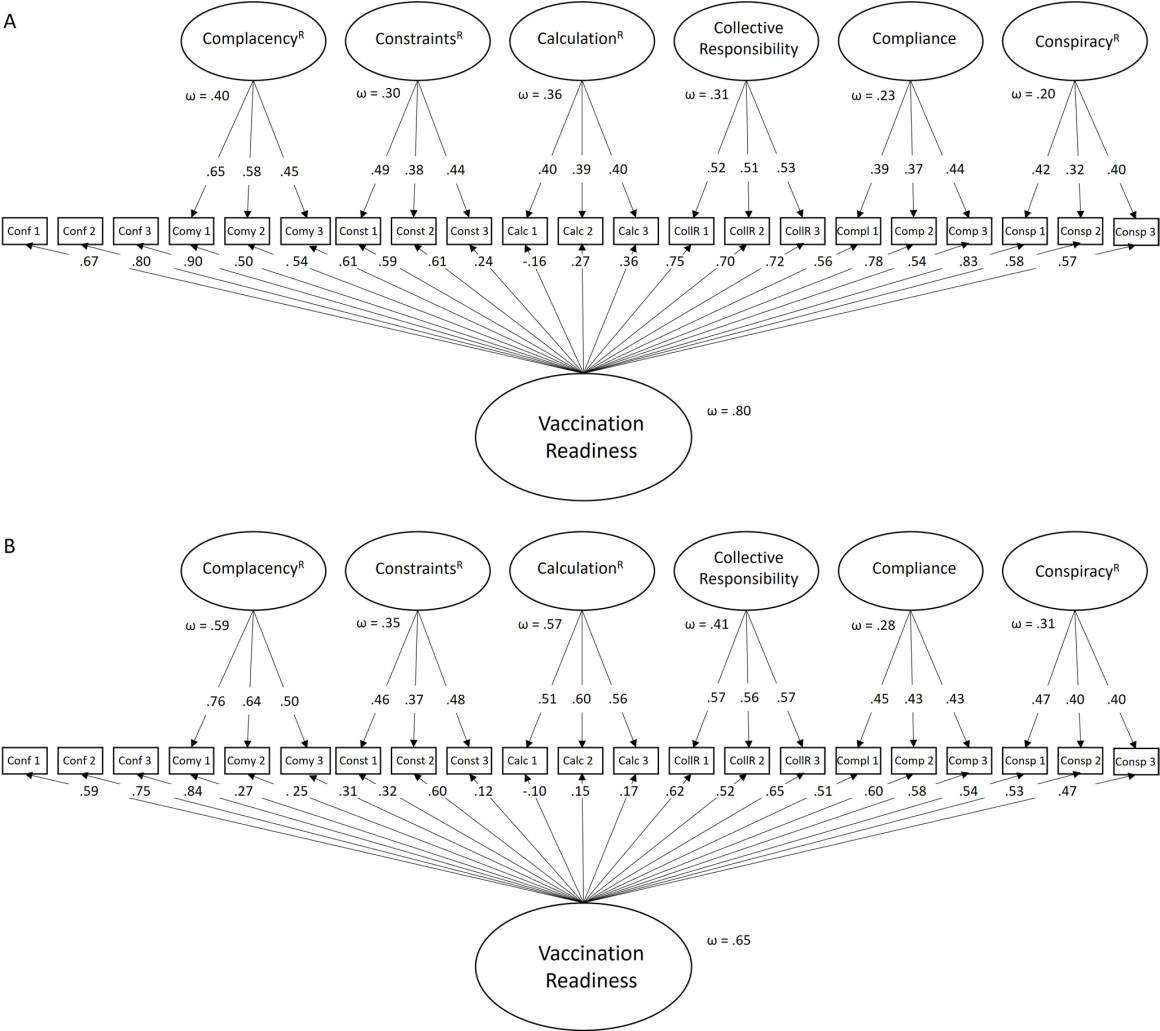
Bifactor confirmatory factor analysis of the 21-item 7 C scale. Loadings are standardized, and loadings that are not significant at α = 0.05 are shown as dotted lines. The subfactors are nested factors, representing what is specific to the component after controlling for general vaccination readiness. conf, confidence; comy, complacency; const, constraints; calc, calculation; collR, collective responsibility; compl, compliance; consp, conspiracy. **A**: German sample, **B**: Greek sample

As suggested by Geiger et al. (2022), the 21-item 7 C scale was modeled as a bifactor model with all items loading on the general factor of vaccine readiness and six orthogonal factors of all components except confidence, which serves as a reference [34]. The bifactor models are depicted in Fig. 3A for the German version and Fig. 3B for the Greek version.

The model fit the data well, the fit indices ranged from acceptable in the Greek bifactor model (χ^2^(168, *N* = 257) = 423.400, *p* <.001, CFI = 0.880, TLI = 0.850, RMSEA = 0.077, SRMR = 0.0625) to good in the German model (χ^2^(168, *N* = 319)) = 470.397, *p* <.001, CFI = 0.921, TLI = 0.901, RMSEA = 0.075, SRMR = 0.0490). The saturation of the general factor was acceptable in the Greek model to large in the German model, with insufficient to acceptable saturation regarding the specific factors.

**Fig. 4:**
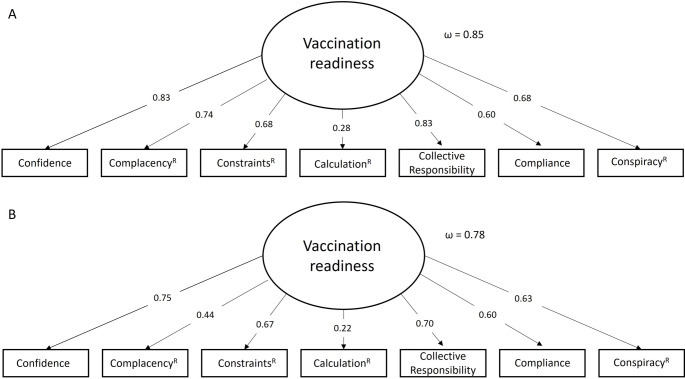
G-factor confirmatory factor analysis of the 7 C scale short version, using one item for each component. **A**: German sample, **B**: Greek sample

The g-factor model for the 7-item short version showed for both samples good fit indices, with χ^2^(15, N = 319) = 34.625, *p* =.003, CFI = 0.976, TLI = 0.967, RMSEA = 0.064, SRMR = 0.0383 and for the German sample (Fig. 4A) and χ^2^(15, N = 257) = 27.390, *p* =.026, CFI = 0.969, TLI = 0.957, RMSEA = 0.057, SRMR = 0.0383 for the Greek sample (Fig. 4B). The factor saturation was acceptable to good for the general factor as well as for all items, except for the component *calculation*^*R*^.

#### Construct Validity

The results of the know-groups method, as applied to age (i.e., younger than 60 years or 60 years and older), working with older people, and the intention to vaccinate against influenza or COVD-19, are shown in Table 5. In the German sample, 240 participants were under the age of 60, 45 participants were working with older people. Furthermore 185 participants indicated their willingness to vaccinate against influenza, while 221 participants expressed their willingness to vaccinate against COVD-19. In the Greek sample, there were 235 participants under the age of 60, 57 participants were working with older people, 170 participants indicated their willingness to vaccinate against influenza, and 155 participants indicated their willingness to vaccinate against the novel coronavirus. The findings indicate that both the 21-item and 7-item versions of the C7 scale can distinguish between the examined groups. However, the results demonstrate no significant difference between the younger and older participants in the Greek sample.

**Table 5:** Known-groups analyses for the 21-item and the 7-item C7 scale

	Age		Working with older people		Intention to vaccinate against influenza		Intention to vaccinate against COVID-19		Significance
	< 60	≥ 60	yes	no	yes	no	yes	no	
21-item C7 German	4.75	5.05	5.21	4.76	5.29	3.86	5.34	3.38	Age* Influenza** COVID-19** working with older people*
21-item C7 Greek	4.64	4.79	4.87	4.59	4.97	3.90	5.05	3.76	Influenza** COVID-19** working with older people*
7-item C7 German	4.49	4.84	4.99	4.51	5.10	3.51	5.14	2.99	Age* Influenza** COVID-19** working with older people*
7-item C7 Greek	4.38	4.64	4.61	4.34	4.75	3.58	4.85	3.44	Influenza** COVID-19** working with older people*

T-Tests were used for all analyses. The mean values of the scales (range 1–7) are presented. *p <.05, ** *p* <.001. Only “yes” and “no” answers were used for the “Intention to vaccinate” groups, “Don’t know” answers were counted as missing values

#### Correlations of the 7 components of the vaccination readiness with socio-demographics and willingness to be vaccinated against measles, influenza, and COVID-19

**Fig. 5:**
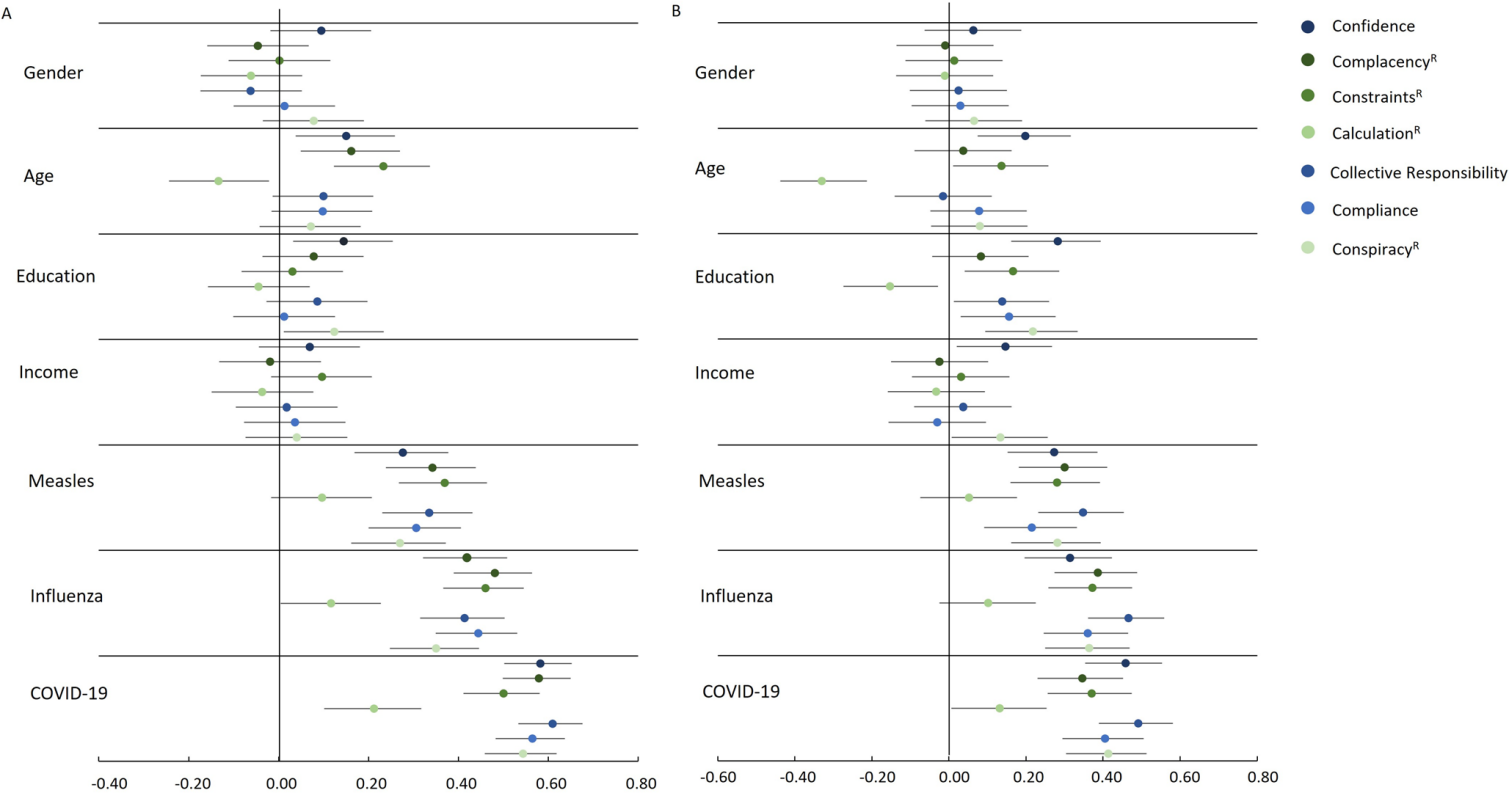
Spearman correlations of the 7 components of vaccination readiness and the willingness to be vaccinated against measles, influenza, and COVID-19. The correlations are shown with the 95% confidence intervals. 1 = women, 2 = men, vaccinations: 0 = “I don’t know” and “no”, 1 = “yes”. **A**: German sample,** B**: Greek sample

Figure 5A (German sample) and Figure 5B (Greek sample) show the relationship between socio-demographic variables, intention to get vaccinated against measles, influenza, and COVID-19, and the individual components of the 7 C model. Age shows statistically significant correlations with vaccination attitudes: Older people have more *confidence* in health authorities, they are less concerned with the individual cost-benefit *calculation*^*R*^ and more concerned with receiving all vaccinations (high values in *constraints*^*R*^). In the German sample, age was also positively correlated with *complacency*^*R*^, indicating that older participants reported a stronger motivation to avoid illness. In the Greek sample, this association did not attain statistical significance.

Education showed a significant correlation with *confidence* in health authorities, as well as with *conspiracy*^*R*^, suggesting that people with higher levels of education are less likely to believe conspiracy theories. In the Greek sample, significant correlations were also observed between education and *constraints*^*R*^, *collective responsibility*, and *compliance*. No significant correlations were found between income and any of the assessed variables in the German sample, while the Greek sample demonstrated significant correlations between income and *confidence* as well as *conspiracy*^*R*^. These findings suggest that individuals with higher incomes exhibit greater confidence in health authorities and are less inclined to adhere to conspiracy theories.

In terms of vaccination readiness, similar trends can be seen for all three vaccinations – measles, influenza and COVID-19 – with *calculation*^*R*^ not being significant in the case of measles and influenza vaccination in both samples. A strong positive correlation exists between *collective responsibility* and *confidence* for the COVID-19 vaccination. The component *confidence* is significantly stronger correlated with COVID-19 vaccination than with the other two vaccination types. In summary, the general readiness to be vaccinated increases mainly with age and education, and shows some vaccination specific differences.

#### Predictive value of vaccination readiness

Binary logistic regressions were performed to further analyze the predictive value of the C7 scale and its individual components.

Participants were asked if they would be vaccinated against measles, influenza, and/or COVID-19. First, a binomial logistic regression was calculated to examine the extent to which vaccination readiness contributes to being vaccinated against measles, influenza, or COVID-19 while controlling for age. In a second step, the seven components of the 7 C scale were included in the logistic regression to examine the predictive value of the single components. Table 6 displays the results.

**Table 6:** Binary logistic regression to compare the explanatory value of the 7 C scale in predicting the intention to vaccinate against measles, influenza, and COVID-19

	Measles						Influenza						COVID-19					
	German			Greek			German			Greek			German			Greek		
Item/Scale	B	Se	OR	B	Se	OR	B	Se	OR	B	Se	OR	B	Se	OR	B	Se	OR
Model 1																		
21-item 7 C scale	**1.028**	**0.152**	**2.795**	**1.066**	**0.192**	**2.904**	**1.200**	**0.159**	**3.320**	**1.485**	**0.214**	**4.413**	**2.469**	**0.292**	**11.807**	**1.800**	**0.244**	**6.051**
Age	**− 0.024**	**0.009**	**0.976**	−0.14	0.011	0.9865	**0.039**	**0.008**	**1.040**	**0.026**	**0.011**	**1.026**	**0.039**	**0.012**	**1.039**	0.004	0.011	1.004
Constant	−2.072	0.679	0.126	−3.052	0.929	0.047	−7.078	0.892	0.001	−7.028	1.081	0.001	−12.129	1.509	0.000	−7.998	1.171	0.000
Nagelkerke´s R^2^	0.279			0.207			0.411			0.350			0.665			0.407		
Model 2																		
Confidence	0.073	0.183	1.076	0.236	0.181	1.266	0.207	0.154	1.229	− 0.145	0.180	0.865	**0.490**	**0.206**	**1.632**	**0.378**	**0.180**	**1.459**
Coymplacency^R^	**0.416**	**0.190**	**1.515**	0.264	0.173	1.302	**0.670**	**0.176**	**1.954**	0.328	0.169	1.389	**0.469**	**0.209**	**1.599**	0.153	0.174	1.166
Constraints^R^	**0.660**	**0.187**	**1.935**	0.346	0.189	1.413	**0.336**	**0.153**	**1.399**	0.186	0.190	1.204	0.014	0.208	1.014	0.087	0.197	1.091
Calculation^R^	− 0.041	0.144	0.959	− 0.118	0.152	0.889	0.087	0.132	1.091	0.164	0.150	1.178	**0.432**	**0.213**	**1.540**	0.104	0.149	1.110
Collective Responsibility	0.100	0.163	1.105	0.099	0.184	1.104	− 0.108	0.143	0.898	**0.523**	**0.190**	**1.687**	**0.611**	**0.187**	**1.843**	**0.509**	**0.199**	**1.663**
Compliance	− 0.008	0.179	0.992	0.025	0.148	1.025	**0.281**	**0.136**	**1.324**	0.235	0.150	1.264	0.199	0.183	1.221	0.211	0.146	1.235
Conspiracy^R^	− 0.135	0.202	0.874	0.170	0.170	1.186	− 0.166	0.181	0.847	0.210	0.172	1.234	0.284	0.223	1.329	0.230	0.176	1.258
Age	**− 0.033**	**0.010**	**0.967**	**− 0.026**	**0.013**	**0.975**	**0.037**	**0.009**	**1.038**	**0.033**	**0.013**	**1.034**	**0.041**	**0.012**	**1.041**	0.001	0.012	1.001
Constant	−2.382	0.867	0.092	−2.961	1.059	0.052	−7.612	1.051	0.000	−7.959	1.255	0.000	−12.677	1.709	0.000	−7.624	1.260	0.000
Nagelkerke´s R^2^	0.339			0.238			0.449			0.380			0.677			0.423		

Bold coefficients are significant at a *p* <.05

Model 1 shows the lowest predictive value for measles, a higher value for influenza, and the highest for the COVID-19 vaccination, with odds ratios of 11.807 and 6.051 in Germany and Greece, respectively.

In the second model, the amount of variance explained was higher in the German sample for all three types of vaccination and was lowest for measles and highest for COVID-19. *Confidence* was a predictor only for COVID-19 vaccination, while *complacency*^*R*^ was a predictor for all types of vaccination in the German sample. *Constraints*^*R*^ was a predictor for measles and influenza, *calculation*^*R*^ for COVID-19, and *compliance* for influenza in the German sample. *Collective responsibility* was a predictor for COVID-19 in both samples and for influenza in the Greek sample. Age was a predictor of vaccination behavior for all types of vaccine except the one for SARS-CoV-2 in the Greek sample. Younger people were more likely to be vaccinated against measles, while older people were more likely to be vaccinated against influenza and SARS-CoV-2. Thus, the pattern of relevant predictors seems to vary between different vaccinations and between countries.

## Discussion

The objective of this study was twofold: first, to validate the German and Greek versions of the 7 C scale of vaccination readiness, and second, to examine differences in vaccination attitude between Germany and Greece. Overall, the scale shows good results in both samples in terms of internal consistency and construct validity, with some weaknesses of items in the *calculatio*n^*R*^ component. The model fit was acceptable to good for the 21-item scales and good for the short form with seven items, as indicated by the CFA.

In general, the German sample was more willing to be vaccinated than the Greek sample, as was apparent in the survey of vaccination status. Notably, a high proportion of the Greek sample responded “I don’t know” when asked about their vaccination status, and significantly fewer participants expressed a willingness to be vaccinated against SARS-CoV-2. The greatest differences between the samples were observed in the *confidence* and *conspiracy*^*R*^ components.

### Vaccination Status

Overall, the study revealed differences in vaccination status between the German and Greek samples. Participants in the Greek sample often answered “I don’t know” when asked if they had been vaccinated against a particular pathogen. The recurrent “I don’t know” responses observed in the Greek sample concerning vaccination history may signify a more extensive problem of inadequate awareness and documentation of personal vaccination records. As evidenced by the qualitative responses, a significant proportion of the Greek participants lacked a certificate of vaccination, relying instead on records maintained by their parents or healthcare providers. These findings suggest that the “I don’t know” responses could be indicative of systemic issues, such as inadequate record-keeping and limited access to personal health information, partly due to the underdeveloped primary healthcare system in Greece that has only undergone significant changes over the past 12 years [56]. In contrast, participants in the German sample who did not know if they had been vaccinated said they did not have their International Certificate of Vaccination or Prophylaxis, also known as the Yellow Card, readily available. This document has been mandatory in Germany since 1961 under the Federal Epidemics Act [57].

The reported vaccination rates for the measles vaccination in this study were almost the same in both countries at 82.1 and 80%, respectively. These rates fall in the lower range for Germany. According to the Robert Koch Institute, 92% of children born in 2017 have been vaccinated twice against MMR [58]. As vaccination has been compulsory in Germany since March 2020 for all persons born after 1970, and no communal facilities such as kindergartens or schools may be attended without vaccination [59], it is expected that the vaccination rate of 95% advised by the WHO, which is necessary for eradication of the virus, can soon be achieved [60]. The lower vaccination rate in the present study can be explained by the fact that the proportion of participants who have already reached retirement age is relatively high in the German sample. Some of the study participants also stated that they were not vaccinated because the vaccine was not yet available during their childhood.

In Greece, vaccination against MMR is only recommended [61]. A study of dental students from 2022 showed that only 56.5% of participants were fully vaccinated [62]. However, another study has shown that while the vaccination coverage rate of first graders is 98.9%, only 83.3% have received a second dose of the vaccine [63], which is well below the WHO target of 95% required for herd immunity [64]. A measles outbreak in Greece in 2017/18 was caused by the low vaccination rate of Roma children, only 8% of whom had received two doses of the vaccine [63].

Although vaccinations against tetanus, diphtheria, and pertussis are mandatory in Greece, but not in Germany [61], the vaccination rates for diphtheria and tetanus in the German sample are significantly higher than in the Greek sample, and up to a third of the Greek participants did not know whether they had been vaccinated. According to the Robert Koch Institute, although the vaccination rate for infants for the first vaccination against diphtheria, tetanus, and pertussis in Germany is 96%, only 77% have received full protection in Germany [58]. In addition, only around half of adults have their vaccination status against tetanus and diphtheria refreshed every 10 years as recommended [65]. There are vaccination gaps, particularly among older people and adults of low socio-economic status living in western Germany [66].

Of the dental students in Greece, only 63.2% stated that they had been vaccinated against tetanus and diphtheria, 47.8% against pertussis [62], which represents a significantly lower proportion than in the present study. A study by Papagiannis et al. (2024) showed that adults in Greece do not have complete immunity to tetanus and diphtheria. Only 59.5% were seropositive against tetanus and 31.5% against diphtheria [67]. A study examining the vaccination coverage of people receiving outpatient psychiatric services in a hospital in Crete found a coverage rate of only 11% [68]. Another study conducted in Greece showed a low seropositivity rate for diphtheria and pertussis in pregnant women. In addition, less than 2% were vaccinated with Tdap, although this was recommended by the Greek national immunization program. The study also showed a correlation between education and antibodies against tetanus. Women with a higher level of education had higher antibody titers against tetanus than women with a lower level of education [69].

The high proportion of Greek study participants who are vaccinated against hepatitis A is probably due to the recurring outbreaks of infections, mainly among Roma [70]. A hepatitis A virus vaccine was first approved in 1999. Until 2008, vaccination was officially recommended only for high-risk groups in the population. However, pediatricians advised vaccinating children at their parents’ expense [71], as there were repeated cases in the general population as well as outbreaks among the Roma population. In 2008, the vaccination was included in the routine national vaccination program for all children over 12 months of age and is fully reimbursed [72]. This makes Greece the only European member state to include hepatitis A vaccination in its national vaccination program, increasing the vaccination coverage of 6-year-old children from 37% in 2006 [71] to 64.5% in 2010 for children aged 0–12 years [73].

A dramatic increase in invasive meningococcal infections, particularly meningococcal C, in Greece in the late 1990s may explain the difference in vaccination status regarding meningococcal C vaccination [74]. As a result, the meningococcal C conjugate vaccine has been used by 72% of pediatricians in Greece since 2001, even though there was no official recommendation for its administration to infants [75] and the vaccination was only included in the national vaccination program in 2005. This has contributed to a significant decrease in the incidence of invasive meningococcal infections in all age groups and, especially, in infants (by 73% between 2006 and 2016), and may also have led to a higher awareness of this vaccination in Greece than in Germany.

The higher vaccination rate for tick-borne encephalitis (TBE) in Germany is probably due to the differences in incidence between these countries [76]. Many regions in Germany are considered high-risk areas for TBE [77], whereas the risk in Greece varies from no risk to a low risk depending on the area [78]. Nevertheless, vaccination rates in Germany’s risk areas are also relatively low and fluctuate greatly, according to the Robert Koch Institute. Nationwide, the vaccination rate in 2020 was, therefore, only around 19% [79].

The influenza vaccination rate was almost the same in both samples, which can be explained by the fact that both Germany [80] and Greece [81] have a vaccination recommendation for people over 60 years, as well as for high-risk patients. On the other hand, there is a significant difference in vaccination behavior with regard to COVID-19 vaccination, with more people willing to be vaccinated in the German sample. Not only have more people been vaccinated, but significantly more people have stated that they have been vaccinated 3 times or more, i.e., are fully vaccinated. The vaccination rate of 78.4% with regard to booster vaccination in the German sample was also significantly higher than the vaccination rate of 62.7% reported by the Robert Koch Institute [82], which can be explained by the age of the sample. The vaccination rate of the Greek sample of booster vaccinations for COVID-19 in our study (56%) is largely consistent with the reported rate of booster vaccinations (58.2%) [83].

### Validity of the 7 C Scale

The 21-item version of the scale showed good internal reliability for the general factor, but the reliability was significantly smaller for the specific factors, especially for the component *calculation*^*R*^. Geiger et al. (2022) came to similar results in the original publication with a Danish sample, explaining the low values of the specific factors with the nested structure and the strong general factor [34]. The version of the scale adapted for COVID-19 also showed similar values [84], while the Japanese version achieved significantly higher values for the specific factors, with the exception of the factor *calculation*^*R*^ [85]. The authors write in the methods section, “McDonald’s omega was calculated for each component and for all items to evaluate internal consistency“ [85]. This could mean that in this study, internal reliability was calculated separately for the individual components but not in the bifactor model, which could explain the high values compared to all other validation studies of the 7 C scale.

The acceptable to good model fit for the 21-item 7 C scale in our study is comparable to the validation studies of the Danish [34], Japanese [85], and the COVID-19-adapted versions [84].

The 7-item version of the 7 C scale was well represented by the general factor model and showed similar values in all validation studies as in the German and Greek versions of our study. The saturation was good, but there was a weakness in the item that represents the component *calculation*^*R*^ and an acceptable to good model fit [34, 84, 85].

The German and the Greek sample differ primarily in the components *confidence*,* complacency*^*R*^, and *conspiracy*^*R*^, with significantly higher values in the German sample. Rees et al. (2022), who recruited participants through the German COVID-19 Snapshot Monitoring (COSMO) to validate the childhood vaccination readiness, also used the collected data to calculate the mean values of the seven components when asking participants if they would vaccinate themselves against COVID-19. The mean values are similar to those of our German sample, with even higher values for the *confidence* component, while the values for the *complacency*^*R*^ and *conspiracy*^*R*^ components were slightly higher in our study [84]. Machida et al. (2024), who examined the vaccination readiness and their patterns in Japan using the 7 C scale, found significantly lower values for all the seven components using the short form of the scale [86]. Geiger et al. (2022), who validated the 7 C scale of vaccination readiness within a Danish sample, did not report mean values [34]. Finally, Kyrianidou et al. (2023), who conducted a cross-cultural study in six European countries, namely Cyprus, France, Germany, Italy, Poland, and Spain, reported mean values far above those of the other studies [87]. As the samples for Germany and France, with 37 and 13 participants, respectively, were too small for the analyses, the median was only collected for the remaining four countries, with the median for all countries for the *complacency*^*R*^ component being 7. The significantly higher overall values may be due to the fact that the data collection took place between December 2020 and January 2021, i.e., in the middle of the coronavirus pandemic, when many countries had imposed lockdowns.

By comparing the histograms of the short form 7 C scale between Germany, Greece, and Japan, it becomes clear why the mean value in Japan is significantly lower than that in Greece and Germany [86]. While most people in Japan answered “4” to the question “I am convinced the appropriate authorities only allow effective and safe vaccines”, most participants in Greece answered “5” on a scale of 1 (strongly disagree) to 7 (strongly agree) and in Germany “6”. The study by Rees et al. (2022) also achieved a significantly higher value of 5.621 for this item of the *confidence* factor than for the other two items, which together form the *confidence* component in the 21-item scale [84] However, in their study, they used the version adapted to COVID-19, i.e., the statement referred directly to the COVID-19 vaccine. The histograms for Japanese and Greek samples were similar for the *complacency* item “I get vaccinated because it is too risky to get infected”, whereas the German sample most frequently answered “strongly agree”. In the study by Rees et al. (2022), which again referred to the COVID infection, a mean value of 4.912 was achieved for this item, while the other two items, which provide additional information on complacency in the 21-item scale, achieved significantly higher values [84]. Further differences can be seen between the countries in the item for *calculation*^*R*^ “I only get vaccinated when the benefits clearly outweigh the risk”. While the modal value in the Japanese study is clearly 4, the participants in the German and Greek samples strongly agreed with this statement, which led to values of 2 and 1 respectively for this reverse-coded item. The study by Rees et al. (2022) showed a mean value for this item that was significantly higher than the values of our study [84], which may be related to the fact that the data collection took place in May 2021 in the middle of the corona pandemic, at a time when it was still assumed that herd immunity would be possible with a vaccination rate of at least 80% [88]. The item that depicts the *collective responsibility* component in the 7-item version, “I see vaccination as a collective task against the spread of diseases”, achieved ceiling effects in all studies; only in the Japanese study was the modal value at 4. The item that depicts the *compliance* component, “It should be possible to sanction people who do not follow the vaccination recommendations by health authorities”, met with great rejection in our German sample, a result that was also shown in the study by Machida et al. (2024) in a Japanese sample, whereas the Greek sample had a modal value of 4, similar to the study by Rees et al. (2022). Here, too, the result of the study by Rees et al. (2022) may be due to the fact that at the time of the survey, discussions were already underway on the introduction of a facility-based mandatory vaccination for certain occupational groups in the health and care sector, which came into force in Germany on March 16, 2022 and was valid until December 31, 2022 [89]. These large differences between the countries were also observed for the *conspiracy*^*R*^ item “vaccinations cause diseases and allergies that are more serious than the diseases they ought to protect from”. While the participants in the German sample strongly rejected this item, the Greek sample also tended to give negative answers, whereas the Japanese sample had a modal value of 4. The participants in the COVID-19 adapted version of the 7 C scale by Rees et al. (2022) also disagreed with this item [84].

The item analysis in our study showed that the *calculation*^*R*^ component, in particular, exhibited weaknesses in all three items, as did item 9, which belongs to the *constraints*^*R*^ component. Some of these items correlated negatively with all other items, and item 10 in the German sample also correlated with the overall scale. Schulz et al. (2024) also found negative correlations for the *calculation*^*R*^ component [31]. This is also reflected in the bifactor CFA, where the factor loading from the general factor “Vaccination readiness” to item 10, “I get vaccinated when I do not see disadvantages for me” (reverse coded), is negative. This means that this item was rated higher by those who consider vaccination from a cost-benefit perspective. The Japanese study also came to the same conclusion, with all three items of the *calculation*^*R*^ component showing negative loadings, which was confirmed in the 7-item version [85]. The original study with a Danish sample did not show negative loadings, but a loading close to zero [34], while the same group conducted two studies with a COVID-19 adapted form of the questionnaire, in which item 9, 10, and 11 each had negative values in one of the two studies, as did the item of the 7-item version in one of the two studies [84]. The authors point out that the loadings fluctuated during the pandemic, which is why they refrained from modifying items. In our study, the modal value was 6 for the German and 7 for the Greek samples. Since vaccinations generally have advantages and the advantages outweigh the disadvantages, it is not surprising that a large proportion of participants agreed with this statement, which does not necessarily indicate a lower willingness to be vaccinated. The findings, which have been consistent across diverse cultural contexts, underscore the necessity to reformulate or revise the theoretical construct of the component *calculation*^*R*^ to enhance its psychometric consistency. Consequently, further theoretical research is necessary to reconsider this component and determine whether it should be definitively incorporated into the 7 C model. In the COVID-19 adjusted scale, the mean value was slightly higher [84], which may be related to the fact that the question here was not generally about a vaccination but specifically about the COVID-19 vaccination, which was the focus of discussion at the time of data collection with all its advantages and disadvantages.

### Predicting Vaccination Behavior

As the 7 C scale measures general readiness to be vaccinated, the second aim of the study was to investigate the extent to which general vaccination readiness can predict the intention to be vaccinated against certain diseases. To this end, we asked the participants whether they would get vaccinated against measles, influenza, or COVID-19. While the pattern of relevant predictors seems to vary between the different vaccinations, the 7 C scale showed the highest predictive value for COVID-19 vaccination, followed by influenza vaccination, and the lowest value for measles vaccination in both countries, with odds ratios for measles and influenza vaccination differing only slightly between the two countries, whereas for COVID-19 vaccination the odds ratio of the German sample was almost twice as high. When analyzing the individual components in more detail, *collective responsibility* was the most important component for COVID-19 vaccination in both the German and Greek samples, followed by *confidence*. This is consistent with other studies [33, 84, 90]. In the German sample, both *complacency*^*R*^ and *calculation*^*R*^ proved to be significant predictors, as did the age of the respondents, with older respondents exhibiting a greater propensity to desire vaccination, while age had no impact in the Greek sample. 

*Collective responsibility* was the only significant predictor of influenza vaccination in the Greek sample, whereas *complacency*^*R*^ was the strongest predictor in the German sample, followed by *constraints*^*R*^ and *compliance*. Age was a significant predictor of influenza vaccination in both samples, which is consistent with the vaccination recommendation in both countries, which recommends annual influenza vaccination primarily for people aged 60 years and older [80, 81]. Similarly, Schulz et al. (2024) observed the vaccine-specific differences [31].

As also stated by Rees et al. (2022), so far, little is known about the relationship between vaccination readiness, vaccination knowledge, and vaccination decision [84]. The qualitative data collected in our study suggests that the participants who were most strongly opposed to vaccination lack of knowledge about how vaccines work, especially the new mRNA vaccines that have been used in the fight against the coronavirus pandemic. Therefore, further studies are needed that examine not only vaccination readiness but also knowledge about the effectiveness of vaccination and vaccination myths in order to better understand the role that knowledge plays in the decision for or against vaccination.

### Limitations

The study has some limitations. Although we made efforts to ensure a diverse sample by including individuals from different social groups and encouraging their social circles to participate in the study, the two samples have certain differences regarding their socio-demographics. When comparing the socio-demographic data of our study with the general population in Germany and Greece, it appears that our sample is likely to have a higher level of education. This may indicate a potential bias that should be taken into account. This bias is a known problem that may be due to the lower participation of individuals with lower levels of education in scientific projects. Furthermore, the recruitment channels we used were mainly aimed at people with a particular interest in research projects.

The overrepresentation of women in our sample is also a significant source of bias. Prior studies have demonstrated an association between being female and lower vaccination willingness [91, 92]. Therefore, gender bias in the sample may have led to an underestimation of overall vaccination willingness as measured by the 7 C scale, limiting the generalizability of our findings to more gender-balanced populations.

It is also important to consider the limitations of the online survey format. Online surveys tend to attract respondents who possess either a high level of technological proficiency or a considerable amount of leisure time, which can lead to potential selection bias and skewed results. In addition, the lack of personal interaction that occurs in face-to-face interviews limits the possibility of obtaining more detailed or nuanced responses. Finally, technical difficulties such as slow loading times or problems with the survey software can frustrate respondents and potentially affect response rates.

Therefore, for future projects, we recommend larger samples and an expanded recruitment plan. Convenience samples tend to be statistically biased, as they consist mainly of WEIRD (Western, Educated, Industrialized, Rich, and Democratic) individuals. As a result, the ability to generalize and make cross-cultural comparisons is limited.

## Conclusion

The present study was able to show that the 7 C vaccination readiness scale is a valid tool in both German and Greek translations and can be utilized in studies of the seven components of vaccination acceptance. Only the *calculation*^*R*^ component showed weaknesses and should be further investigated or adapted in future studies. It was shown that there are culture-specific differences between the German and Greek samples, which mainly concern the components *confidence*, *complacency*^*R*^, and *conspiracy*^*R*^. Since knowledge about vaccinations and misinformation can play an important role in vaccination decisions, future studies should examine knowledge about vaccinations and their effects, as well as knowledge about vaccination myths, in addition to the 7 C scale and the vaccination status.

## Supplementary Information

Below is the link to the electronic supplementary material.

Supplementary File 1 (DOCX 23.4 KB)

## Data Availability

The datasets presented in this study can be found in the online repositories: OSF (https://osf.io/ynhe4/).

## Abbreviations

CFA: confirmatory factor analysis
CFI: comparative fit index
COSMO: COVID-19 Snapshot Monitoring
MMR: measles, mumps, and rubella
OSF: Open Science Framework
RMSEA: root mean square error of approximation
SRMR: standardized root mean square residual
TBE: tick-borne encephalitis
TFI: Tucker-Levis index
WEIRD: Western, Educated, Industrialized, Rich, and Democratic
WHO: World Health Organization
