# Supplementary material for: The 7 C Vaccination Readiness Scale: An Empirical Country Comparison Between Germany and Greece
Source: J Epidemiol Glob Health. 2025 Nov 21;15(1):136. doi: 10.1007/s44197-025-00485-9 (PMC12638586; doi:10.1007/s44197-025-00485-9)
Supplement: Supplementary file 1 — (DOCX 23.4 KB) [file 44197_2025_485_MOESM1_ESM.docx]

Appendix 1

|  | **German sample**  **(n = 319)** | **Greek sample**  **(n = 257)** | ***p***^1^ |
| --- | --- | --- | --- |
| **Measles, mumps, rubella (MMR)**  **(n,%)**  Yes  No  I don't know | 262 (82.1)  40 (12.5)  17 (5.3) | 208 (80.6)  22 (8.5)  27 (10.5) | **0.02** |
| **Influenza (n, %)**  Yes, one time  Yes, several times  Yes, every year  No  I don't know | 33 (10.3)  84 ( 26.3)  72 (22.6)  113 (35.4)  17 (5.3) | 40 (15.6)  53 (20.6)  58 (22.6)  90 (35.0)  16 (6.2) | 0.266 |
| **COVID-19 (n. %)**  Yes  No  one time  two times  three times  four times  five times  six times | 292 (91.5)  27 (8.5)  4 (1.3)  38 (11.9)  144 (45.1)  61 (19.1)  33 (10.3)  12 (3.8) | 222 (86.4)  35 (13.6)  7 (2.7)  71 (27.6)  111 (43.2)  19 (7.4)  7 (2.7)  7 (2.7) | 0.073  **< 0.001** |
| **Rotaviren (n, %)**  Yes  No  I don't know | 47 (14.7)  113 (35.4)  159 (49.8) | 42 (15.9)  72 (27.9)  144 (55.8) | 0.165 |
| **Dipftheria (n,%)**  Yes  No  I don't know | 236 (74.0)  17 (5.3)  66 (20.7) | 142 (55.0)  29 (11.2)  86 (33.3) | **< 0.001** |
| **Tetanus (n,%)**  Yes  No  I don't know | 306 (95.9)  5 (1.6)  8 (2.5) | 208 (80.6)  24 (9.3)  25 (9.7) | **< 0.001** |
| **Pertussis (n,%)**  Yes  No  I don't know | 208 (65.2)  50 (15.7)  61 (19.1) | 154 (59.7)  33 (12.8)  70 (27.1) | 0.062 |
| **Poliomyelitis (n,%)**  Yes  No  I don't know | 277 (86.8)  10 (3.1)  32 (10.0) | 158 (61.2)  29 (11.2)  70 (27.1) | **< 0.001** |
| **Hämophilus influenzae Typ b (n,%)**  Yes  No  I don't know | 92 (28.8)  79 (24.8)  148 (46.4) | 66 (25.6)  61 (23.6)  130 (50.4) | 0.578 |
| **Hepatitis B (n,%)**  Yes  No  I don't know | 243 (76.2)  43 (13.5)  33 (10.3) | 175 (67.8)  33 (12.8)  49 (19.0) | **0.012** |
| **Meningococcus B (n,%)**  Yes  No  I don't know | 74 (23.2)  114 (35.7)  131 (41.1) | 92 (35.7)  67 (26.0)  98 (38.0) | **0.002** |
| **Meningococcus C (n,%)**  Yes  No  I don't know | 75 (23.5)  104 (32.6)  140 (43.9) | 95 (35.8)  62 (24.0)  100 (28.8) | **0.001** |
| **Pneumococcus (n,%)**  Yes  No  I don't know | 107 (33.5)  97 (30.4)  115 (36.1) | 97 (37.6)  82 (31.8)  78 (30.2) | 0.334 |
| **Varicella (n,%)**  Yes  No  I don't know | 123 (38.6)  155 (48.6)  41 (12.9) | 144 (55.8)  88 (34.1)  25 (9.7) | **< 0.001** |
| **Human Papillomavirus (n,%)**  Yes  No  I don't know | 79 (24.8)  168 (52.7)  72 (22.6) | 71 (27.5)  125 (48.4)  61 (23.6) | 0.611 |
| **other vaccinations (n,%)**  Hepatitis A  FSME  other  with one other  two other  three other  four other  five other  six other | 36 (11.3)  68 (21.3)  81 (25.4)  57 (17.9)  17 (5.3)  4 (1.3)  1 (0.3)  1 (0.3)  1 (0.3) | 127 ( 49.4)  0 (0.0)  1 (0.4)  1 (0.4)  0 (0.0)  0 (0.0)  0 (0.0)  0 (0.0)  0 (0.0) | **< 0.001**  **< 0.001**  **< 0.001** |
